# Massive simple hepatic cyst triggered sudden cardiac arrest in a middle-aged, female patient due to compression of the right heart

**DOI:** 10.1308/rcsann.2024.0108

**Published:** 2025-04-08

**Authors:** F Dimek, M Williams, K Nelson, JJ French

**Affiliations:** ^1^ Freeman Hospital, UK; ^2^ Royal Victoria Infirmary, UK

**Keywords:** Hepatic cyst, Sudden cardiac arrest, Ventricular fibrillation

## Abstract

Liver cysts are common, often remain clinically silent and need treatment only if symptoms occur. A 65-year-old woman presented with abdominal bloating and swelling due to a massive liver cyst (190×210mm). Urgent surgical treatment (laparoscopic deroofing) was planned. Before the surgical date the patient suffered a nonfatal cardiac arrest, likely due to a cardiac compression by the cyst.

This is a rare case of a liver cyst causing a very serious cardiac adverse event. Pre-event, there were no signs of cardiac decompensation. We want to highlight the importance of early surgical treatment of large liver cysts, even in patients with mild symptoms.

## Background

Simple hepatic cysts are very common in the population, with a prevalence of approximately 2.5–18.0%.^1,2^ Most are asymptomatic and do not require treatment. The presence of symptoms is an indication for treatment or surveillance.^3^ Broadly, there are two treatment options: laparoscopic fenestration or percutaneous aspiration sclerotherapy.^3^

The primary treatment goal of simple hepatic cysts is symptom relief, with volume reduction a secondary consideration.^3,4^

Complications from simple hepatic cysts are rare and include infections, haemorrhage, rupture or compression of adjacent structures such as bile ducts or, rarely, the heart.^5,6^

Compression of the heart may be associated with arrhythmia ranging from the benign to life-threatening. According to previous case reports, mechanical compression of the heart due to larger hepatic cysts or hiatal hernia most commonly cause atrial heart rhythm disorders, complete AV-block or an acute heart failure.^6–9^ Serious cardiac events such as sudden cardiac arrest (SCA) due to compression of the heart have not been described to this date. SCA is defined as ‘sudden cessation of normal cardiac activity with haemodynamic collapse’^10^ and sudden cardiac death (SCD) causes around 15–20% of all death in Western countries.^11^ There are many reasons for the occurrence of SCA: coronary artery disease, primary electric diseases and cardiomyopathies are the most frequent, but myocarditis and coronary abnormalities also play an important role.^10^ Here, we describe a case of SCA likely triggered by an atrial mechanical compression.

## Case history

A 65-year-old white woman presented to her general practitioner with abdominal swelling, bloating and a mass in the right upper quadrant. Notably, the patient denied other symptoms such as pain, nausea/vomiting, jaundice, changes in bowel movement or signs of systemic infection. There were no cardiac symptoms.

Medical history included essential hypertension, bronchial asthma, a left bundle branch block (LBBB) and a mildly impaired left ventricular systolic function. History of smoking was denied by the patient, and she drank 1–2 units of alcohol daily.

There was a family history of postpartum cardiomyopathy in a first-degree relative.

**Figure 1:**
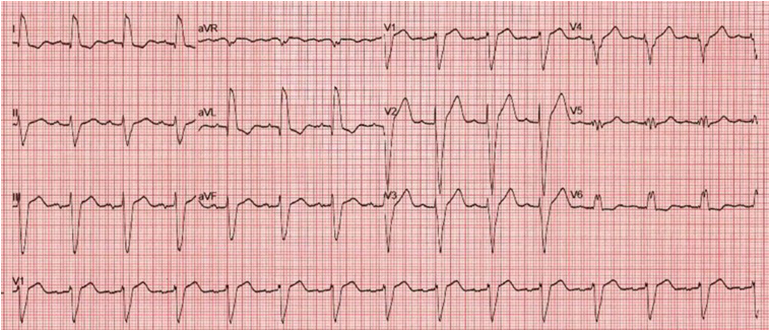
The 12-lead electrocardiogram one hour after cardiac arrest shows a normofrequent sinus rhythm with preexisting left bundle branch block. No other pathologies were detected (paper speed 25mm/s).

Two years pre-event, a computed tomography (CT) coronary angiogram and calcium score for atypical angina pectoris found no cardiac pathology. The CT also revealed liver cysts, which were occupying most of the liver. At that time an echocardiogram showed mildly impaired left ventricular function and an electrocardiogram revealed a LBBB (Figure 1).

At initial consultation in the hepatopancreaticobiliary (HPB) service, the patient was found to be well with a normal body mass index (22.9kg/m^2^). Physical examination revealed a mildly tender abdomen in the right upper quadrant with a palpable liver edge below the costal margin.

An abdominal ultrasound revealed two known anechoic thin-walled cysts noted in the liver, one which had grown to the massive size (219×215×189mm).

**Figure 2:**
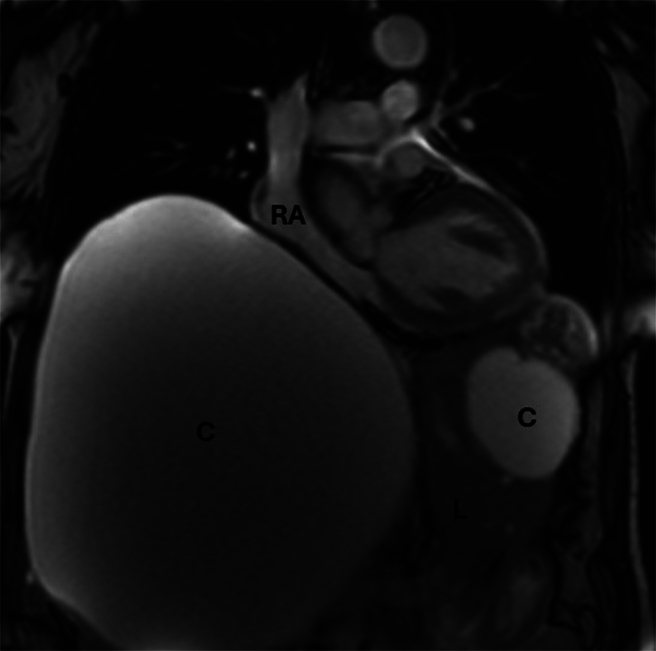
Abdominal MRI revealed two hepatic cysts (C), the massive one (190×210mm) compressing the right atrium (RA) and liver (L). Both cysts appeared as simple hepatic cysts radiologically. MRI = magnetic resonance imaging.

Subsequent contrast magnetic resonance imaging (MRI) showed three smaller cysts alongside the large unilocular cyst in the right lobe (190×210mm), all classified as simple hepatic cysts. Displacement of adjacent structures as well as compression and displacement of the biliary obstruction were caused by the major cyst (Figure 2).

Liver function tests showed increased alanine transaminase (72IU/l (reference 3–40IU/l)), gamma-glutamyl-transferase (298IU/l (reference 8–60IU/l)) and alkaline phosphatase (236IU/l (reference 30–100IU/l)).

Considering the altered liver function and abdominal symptoms, surgical treatment of the large liver cyst by means of robotic or laparoscopic liver cyst deroofing was discussed, agreed with the patient and scheduled on an urgent basis.

However, eight weeks after HPB consultation and before the planned surgical procedure, the patient suffered, at home, an out-of-hospital cardiac arrest. Cardiopulmonary resuscitation was initiated immediately by the patient’s husband and continued until the paramedics arrived approximately 10 minutes later. The initial electrocardiogram showed ventricular fibrillation and, after application of adrenaline and direct current cardioversion applied twice, a return of spontaneous circulation was reached.

At hospital, a pulmonary embolism, pneumothorax or acute intracranial pathology was excluded by CT. An echocardiogram showed mild dilatation of the left ventricle with normal wall thickness and a globally severely impaired ejection fraction (EF) of 20–30%. There were no signs of valve anomalies.

**Figure 3:**
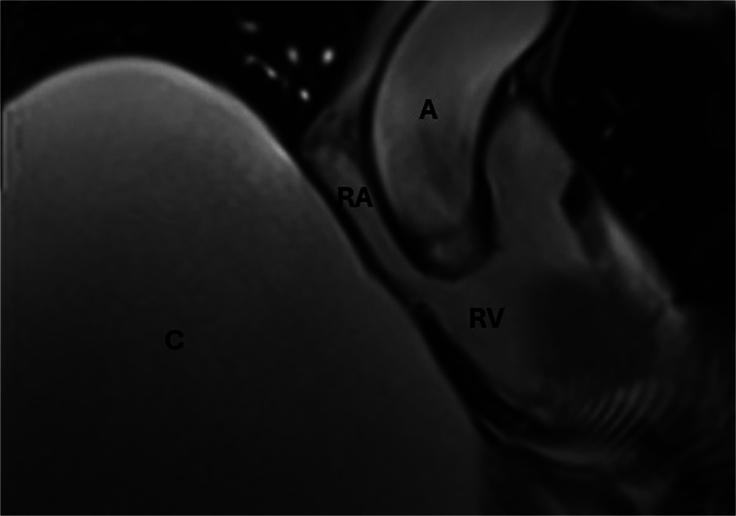
Cardiac MRI reveals the compression of right atrium (RA) and right ventricle (RV) due to a massive hepatic cyst (C). A = aorta; MRI = magnetic resonance imaging.

CT of the coronary arteries did not reveal any flow-limiting coronary artery disease. A cardiac MRI scan revealed a right atrial/ventricular compression throughout the cardiac cycle (Figure 3). It also showed normal gadolinium tissue kinetics and no definite myocardial scar/fibrosis.

The patient was monitored on a coronary care unit and established on guideline-directed medical therapy for heart failure.

Given the radiological findings, obstruction to right ventricular inflow by the liver cyst appeared to be implicated in triggering a cardiac arrest. An ultrasound-guided drain of the massive hepatic cyst was performed to reduce this atrial compression.

A secondary prevention implantable cardioverter defibrillator with resynchronisation therapy (CRT-D) was then implanted via the standard left-sided transvenous approach. This was monitored remotely in accordance with standard practice.

An expedited HPB clinic consultation occurred 40 days after the event. An ultrasound imaging (USS) was performed 12 days after the consultation to check the size of the cyst. Laparoscopic deroofing of the cyst occurred 22 days after the USS.

Following the procedure, the patient has no abdominal or cardiac symptoms with New York Heart Association Class 1 symptoms. CRT-D interrogation reveals no episodes of arrhythmia. Left ventricular function has improved from severely to moderately impaired, EF 40%.

## Discussion

Simple hepatic cysts often remain clinical silent. Treatment is recommended only when symptoms occur.^3,5^ This case highlights the importance of active management and early treatment of simple liver cysts once a critical size is reached (with or without symptoms).

To our knowledge, this is the first reported case of SCA-related compression of the right heart by a liver cyst without prior major cardiac symptoms.

Compression of the heart by abdominal or thoracic masses is not a rare phenomenon. In terms of hepatic cysts, however, most cardiac complications consisted of supraventricular arrhythmias such as atrial fibrillation or flutter^9,12–14^ while hiatal hernias can, rarely, lead to atrioventricular blocks.^7,8^

In all previously reported cases, patients showed symptoms of heart failure or arrhythmia. In this case, the main and serious cardiac symptom that the patient developed was ventricular fibrillation with subsequent cardiac arrest.

The presence of cardiomyopathy in her first-degree relative, combined with the SCA of this patient, is suggestive for the diagnosis of a nonischemic nondilated cardiomyopathy. As nonischemic cardiomyopathies are ordinarily considered lower risk of SCD,^15^ it seems likely that external compression by the liver cyst is at least strongly implicated in the aetiology of her cardiac arrest.

Alternative pathologies causing SCA, such as acute coronary syndrome, long-QT syndrome, congenital heart diseases, or electrolyte imbalance, were ruled out. A further indication that the liver cyst triggered the SCA is the fact that no late gadolinium enhancement was detected, which would increase the risk for the occurrence of major ventricular arrhythmias in nonischaemic cardiomyopathies.^16^

Hence, we advocate early treatment in patients with hepatic cysts, once signs of organ displacement, in particular myocardial compression, are present.

The patient’s life changed dramatically after the cardiac event: it is noted there have been psychological consequences in terms of anxiety after SCA, significantly impacting the patient’s quality of life, specifically, the feeling of constant fear of recurrent SCA and needing company at all times so as not to be left alone. Diminished quality of life and worse cognitive performance is seen frequently in patients after SCA and has been shown previously.^17^

The loss in quality of life is a further indication that treatment of large liver cysts should be considered urgently in order to prevent SCA and detrimental and severe consequences.

## Conclusion

In this case report, we highlight the importance of considering urgent treatment of large hepatic cysts. Major complications such as SCA can occur even in patients with minor to no symptoms. Assessment by ultrasound and MRI should be performed prior to definitive treatment (laparoscopic deroofing). A temporary (percutaneous drain) intervention may be indicated prior to definitive treatment.
